# Induction of Apoptosis with Silver Nanoparticles Obtained Using Thermophilic Bacteria

**DOI:** 10.3390/jfb15060142

**Published:** 2024-05-24

**Authors:** Kotryna Čekuolytė, Diana Šapaitė, Estera Žemgulytė, Renata Gudiukaitė, Eglė Lastauskienė

**Affiliations:** Institute of Biosciences, Life Sciences Center, Vilnius University, Sauletekis Avenue 7, LT-10257 Vilnius, Lithuania; kotryna.cekuolyte@gmc.vu.lt (K.Č.);

**Keywords:** thermophilic bacteria, silver nanoparticles, apoptosis, caspase

## Abstract

Yeasts resistant to antifungals have become an increasing risk to human health. One of the best antimicrobial properties is reported to be present in silver nanoparticles (AgNPs); however, little is known about the antimicrobial potential of AgNPs produced using thermophilic bacteria. How AgNPs cause cell death is different depending on the type of the cell, and the mode of death induced is cell-type specific. Apoptosis, one of the types of regulated cell death, can be extremely useful in the fight against infection because surrounding cells that have phagocytic activity can efficiently absorb the apoptotic bodies formed during apoptosis. In the course of this work, for the first time, comprehensive antifungal studies of AgNPs were performed using thermophilic *Geobacillus* spp. bacteria against *Candida guilliermondii*, also with the addition of the model yeast *Saccharomyces cerevisiae*. The determined minimal inhibitory concentrations (MICs) were 10 μg/mL against *C. guilliermondii* and 50 μg/mL against *S. cerevisiae* for *Geobacillus* sp. strain 25 AgNPs, and for *Geobacillus* sp. 612 the MICs were 5 μg/mL and 25 μg/mL, respectively. It was shown for the first time that the exposure of the yeast cells leads to caspase activation in both *S. cerevisiae* and *C. guilliermondii* after exposure to *Geobacillus* spp. AgNPs. Also, a statistically significant change in the number of cells with permeable membranes was detected. Moreover, it was shown that the antimicrobial effect of the AgNPs is related to ROS generation and lipid peroxidation in *C. guilliermondii* yeast.

## 1. Introduction

Given the growing number of individuals affected by immune-related conditions and aging demographics, it is of utmost importance to prioritize the care of patients with comorbidities [1,2]. Among these individuals, *Candida* infections are alarmingly common and pose significant challenges. Moreover, this vulnerable population often experiences prolonged hospital stays, while *Candida* yeasts, capable of forming biofilms on surfaces, contribute to the contamination of vital medical devices used in patient care [3]. While *Candida albicans* has been extensively studied as a pathogen, the escalating incidence of non-albicans *Candida* infections raises concerns. Particularly, the rapid development of fluconazole and amphotericin B resistance in *Candida guilliermondii* presents a pressing issue [4]. Consequently, the search for novel drugs with antifungal properties becomes imperative, emphasizing the significance of this study.

Silver nanoparticles (AgNPs) offer a promising strategy to address this challenge. Their superior permeability enables them to combat fungi more effectively than traditional antifungal drugs. AgNPs target multiple cellular pathways, inhibiting the growth of susceptible and drug-resistant strains [5,6,7,8]. However, chemical and physical synthesis techniques are highly polluting for the environment. One approach is to employ biological synthesis methods to reduce adverse effects [9]. In the previous works, it was shown that bacteria of the genus *Geobacillus* can synthesize AgNPs [10,11,12]. AgNPs obtained via chemical or physical synthesis are well-described substances used for various antifungal and antibacterial purposes. However, little is known about the antimicrobial effects of AgNPs obtained using thermophilic bacteria. 

Cell death is the irreversible loss of normal cell functions, which is accompanied by inhibition of cell division and loss of homeostasis. If cell death is associated with the activation or inactivation of some signaling pathways, this type of cell death is considered regulated cell death (RCD), since the process is controlled at the gene level [13]. RCD is an induced and programmed cell death, during which the formed apoptotic bodies can be efficiently taken up by neighboring cells with phagocytic activity. For this reason, the induction of apoptosis in skin pathogens can be extremely useful in the fight against infection. The induction of programmed cell death is also one of the favorable mechanisms for eliminating pathogens. During yeast apoptosis, the amino acids, peptides, nucleotides, and lipids, as well as formed apoptotic bodies, are released into the surrounding medium and can be used by the phagocytes as a source for the regeneration of the epithelium tissue [14]. Yeast apoptosis is difficult to analyze since the markers used for the discrimination between apoptosis and necrosis mammalian cells are frequently overlapping. Often, the cytotoxic potential of AgNPs is determined by their physicochemical properties. The concentration of AgNPs, the duration of incubation with cells, and the type of cells exposed also affect the type of cell death and toxicity induced with AgNPs [15,16].

One of the differences that can affect the antifungal effect of AgNPs is the difference in cell wall composition since the cell wall and cell membrane are the primal targets of the AgNPs. *S. cerevisiae* has more glucans in its cell wall compared to *C. guilliermondii*, and *C. guilliermondii* has more mannans compared to *S. cerevisiae* [17,18]. Thus, it is for these reasons that separate studies are needed to investigate the effect of AgNPs on specific microorganisms. During this work, the antifungal activity of AgNPs obtained via *Geobacillus* spp. strains 25- and 612-induced synthesis was tested against the yeasts *Saccharomyces cerevisiae* and *C. guilliermondii*. The yeast *S. cerevisiae* was chosen as the control, it is a model organism with the well-described mechanisms of programmed cell death (membrane permeabilization, caspase activation, formation of the DNA strand brakes, etc.).

This research aims to evaluate the antifungal activity of biosynthesized AgNPs against pathogenic yeast *C. guilliermondii* and model organism *S. cerevisiae*, by assessing apoptogenic capabilities: active caspases, DNA fragmentation, increase in membrane permeability, ROS generation, and lipid peroxidation. Such determination of the mechanism of the antimicrobial effect of *Geobacillus* spp. AgNPs is performed for the first time.

## 2. Materials and Methods

Sources of microorganisms. The *Geobacillus* spp. 25 and 612 strains used in the biological synthesis of AgNPs were isolated in the Department of Microbiology and Biotechnology, Institute of Biosciences (former Faculty of Natural Sciences, Vilnius, Lithuania), Life Sciences Centre, Vilnius University (Lithuania), from an oil well in Lithuania. The antimicrobial effect of the obtained AgNPs was studied against *Saccharomyces cerevisiae* (BY4742) (collection of Microbiology and Biotechnology Departement, Vilnius University, Vilnius, Lithuania) and *Candida guilliermondii*, which was isolated from the skin of an atopic dermatitis patient [19].

AgNPs biosynthesis. The two *Geobacillus* spp. strains (25 and 612) were used for the biosynthesis of AgNPs. The AgNPs were prepared as described in Cekuolytė et al., 2023 [10]. Briefly, cell-free lysates of the aerobically grown *Geobacillus* spp. strains were treated with AgNO_3_ (ROTH, Karlsruhe, Germany) to a final concentration of 2 mM. The mixtures were incubated for 48 h at 55 °C and 200 rpm. After the incubation period, medium components were removed by centrifugation at 3000× *g* for 10 min and AgNPs were collected via centrifugation at 16,000× *g* for 15 min. The obtained pellets were washed three times with 70% ethanol and three times with deionized water.

Characterization of AgNPs. The Zeta potential values analysis and size distribution using the dynamic light scattering method were carried out in the same manner as in Cekuolyte et al., 2023 [10]. The obtained AgNPs were kept in 1 M sorbitol or YPD growth medium for 12 h in this study in order to assess the AgNPs’ size distribution and the Zeta potential values (the AgNPs were washed with distilled water before the measurements).

Determination of the minimal inhibitory concentrations (MIC). The MIC of the AgNPs is determined by using a spot test according to Suppi and co-authors [20]. Yeast cells are grown overnight in the YPD medium (glucose 2% (ROTH, Germany); peptone 2% (ROTH, Germany); and yeast extract 1% (ROTH, Germany)) at 30 °C and 130 rpm till the exponential phase is reached. After that, cells were collected and diluted to 1.5 × 10^7^ and washed three times with 1 M sorbitol. Then, 100 μL of cell suspension is transferred to the 96-well microplate. A total of 100 μL of AgNPs of seven different concentrations (250 μg/mL, 100 μg/mL, 50 μg/mL, 25 μg/mL, 10 μg/mL, 5 μg/mL, and 1 μg/mL) suspended in 1 M sorbitol are added to the wells. As a control, 100 μL of 1 M sorbitol is used. The microplate is incubated at 30 °C 24 h. After incubation, 5 μL of the suspension from each well is transferred to the solid YPD medium. The YPD plates are incubated at 30 °C 48 h. After the incubation the appeared colonies are counted. All the experiments were independently repeated three times.

Induction of cell death. The induction of cell death in this work was performed in two ways: by incubating cells with AgNPs in a yeast growth medium and with sorbitol, which acts as a more neutral solution compared to the growth medium. All cell death experiments were performed independently three times. During induction in a yeast growth medium, cells that have reached the exponential phase of growth are harvested. A total of 1.5 × 10^7^ cells in 1 mL of YPD medium are used for apoptosis induction. The selected concentration of AgNPs is added and incubated at 30 °C. During induction in sorbitol, cells that have reached the exponential phase of growth are harvested. A total of 1.5 × 10^7^ cells are washed three times in sorbitol and centrifuged at 2000× *g* for 5 min. Cells are suspended in 1 mL of sorbitol. Then, 100 μL of the cell suspension is transferred to a new test tube and 100 μL of the selected concentration of AgNPs is added and incubated at 30 °C till further analysis. 

Evaluation of the cell viability. The viability of yeast cells after exposure to AgNPs at different time points (1, 2, 4, 8, and 24 h) is assessed via clonogenic assay. Once the cells have reached the exponential phase of growth, they are harvested. Then, 1.5 × 10^7^ cells are washed three times with 1 M sorbitol and centrifuged at 2000× *g* for 5 min. Cells are suspended in 1 mL of sorbitol. The cell suspension is divided into two test tubes of 500 μL each, and 500 μL of AgNPs of the selected concentration is added (1/10 MIC: 1 μg/mL of *Geobacillus* sp. 25 and 0.5 μg/mL of *Geobacillus* sp. 612 for *C. guilliermondii* and 5 μg/mL and 2.5 μg/mL for *S. cerevisiae,* respectively). As the control, 500 μL of 1 M sorbitol is used. The cell suspension is incubated at 30 °C. After incubation, 100 μL of the cell suspension is plated on a solid YPD medium. The plated yeast cells are grown for 48 h at 30 °C. After the incubation, the CFU is counted. 

Determination of the cell death type. In this work, the type of cell death was assessed using markers characteristic of RCD: active caspases, genomic DNA fragmentation, and membrane permeability. Activated caspases and DNA fragmentation are markers of early apoptosis. Membrane permeability in dying cells occurs due to the primary or secondary necrotic process. Secondary necrosis is often caused by apoptosis, whereas the primary necrotic phenotype (which occurs without any apoptotic features) is commonly attributed to an uncontrolled, random type of cell death [21]. 

Detection of the active caspases. Active caspases in yeast cells are detected using the CaspACE^TM^ FITC-VAD-FMK In Situ Marker kit (Calbiochem, San Diego, CA, USA). After induction of apoptosis, cells are washed three times with 1 × PBS buffer and centrifuged at 2000× *g* for 5 min. After washing, the yeast cells are suspended in 1 mL of 1 × PBS buffer, and 300 μL of the cell suspension is transferred to a new tube. After that, add 1 μL FITC-VAD-FMK and incubate in the dark for 30 min (for *S. cerevisiae*) or 1 h (for *C. guilliermondii*) at 30 °C. After incubation, cells are washed three times in 1 × PBS buffer. Cells are suspended in 100 μL 1 × PBS buffer and fixed with 10% buffered formaldehyde for 30 min at room temperature. After fixation, cells are washed three times. For fluorescence microscopy, cells are suspended in 100 μL 1 × PBS buffer. A drop of the sample is transferred onto a slide covered with poly-L-lysine, covered with a coverslip. Samples are analyzed with a Nikon Eclipse 80i (Nikon, Tokyo, Japan), using a FITC filter (magnification 1000×). For flow cytometry, cells are suspended in 100 μL of 1 × PBS buffer. A sample of 20,000 cells is analyzed. A VU LSC Institute of Biotechnology BD FACSymphony^TM^ A1 flow cytometer is used for flow cytometry experiments (excitation wavelength 488 nm, FITC emission wavelength 535 nm). Flowing Software 2.5.1 is employed for the analysis.

Evaluation of DNA fragmentation. Assessment of DNA fragmentation is performed using the Apoptosis DNA Ladder Assay Kit (Abcham, Cambridge, UK). Two replicates of each experiment are performed after induction of apoptosis (in both yeasts) according to the manufacturer’s protocol. Samples are fractionated on a 1.2% agarose gel (stained with 0.5 μg/mL ethidium bromide). Electrophoresis is carried out under conditions of 5 V/cm for 1.5 h. After electrophoresis, the gel is visualized with a UV transilluminator and photographed. 

Evaluation of the cell membrane permeability. Changes in yeast cell membrane permeability are determined via propidium iodide (PI) staining of the cells. After the exposure of *Geobacillus* spp. strains 25 and 612 AgNPs for 30 min, yeasts cells are washed in 1 × PBS buffer and centrifuged at 2000× *g* for 5 min. Cells are resuspended in 1 mL of 1 × PBS buffer, and 1 μL of PI (1 mg/mL) is added. The yeast cells are incubated with the dye for 10 min at room temperature. After incubation, cells are washed with 1 × PBS buffer and fixed with 10% buffered formaldehyde for 30 min, centrifuged, and resuspended in 1 × PBS buffer. In the case of *C. guilliermondii*, fluorescence is measured at the VU LSC Institute of Biotechnology with a BD FACSymphony^TM^ A1 flow cytometer (BD Biosciences, San Jose, CA, USA) (excitation wavelength 488 nm, emission wavelength 535 nm). A sample of at least 100,000 cells is analyzed. Flowing Software 2.5.1 is employed for the analysis. In the cases of *S. cerevisiae*, a drop of the sample is transferred onto a slide covered with Poly-L-lysine, covered with a coverslip. Samples are analyzed with an Olympus IX83 Inverted Light Microscope (Olympus, Japan).

Detection of the ROS. To test the formation of ROS in the cells exposed to AgNPs alone, cell cultures of *C. guilliermondii* are cultivated in liquid YPD at 130 rpm up until the exponential phase (12–14 h). Centrifugation is used to wash the grown cells twice with deionized water and twice with a 1 M sorbitol solution, and the cells are then suspended in 1 M sorbitol. The cells are treated with AgNPs MICs and incubated at 30 °C for 15 min. Yeast cells of the negative control are treated with no compounds, and positive control yeast cells are treated with 50 μM of Tert-Butyl Hydroperoxide (TBHP) for 4 h. Next, cells are prepared for ROS formation analysis by using a modified DCFDA/H2DCFDA-Cellular ROS Assay Kit (ab113851) (Abcham, UK) protocol. A diluted DCFDA solution is added to the final concentration of 10 µM to stain the cells. Then, cells are incubated for 2 h at 30 °C in the dark. Afterward, cells are washed once with 1 × Buffer. Fluorescence microscopy is performed with a fluorescein isothiocyanate (FITC)-specific filter set using a Nikon Eclipse 80i microscope (Nikon, Japan). ImageJ is employed for quantitative evaluations of fluorescent cell intensity. For flow cytometry, BD FACSCanto^TM^ II flow cytometer (488 nm/535 nm) is used.

Evaluation of lipid peroxidation. Cell cultures of *C. guilliermondii* are cultivated in liquid YPD at 130 rpm up until the exponential phase (12–14 h). Centrifugation is used to wash the grown cells twice with deionized water and twice with a 1 M sorbitol solution, and the cells are then suspended in 1 M sorbitol. *C. guilliermondii* cells are treated with 10 μg/mL (strain 25) and 5 μg/mL (strain 612) of AgNPs in 1 × PBS buffer. For positive controls, Cumene hydroperoxide is added to a final concentration of 100 μM, and for negative controls, no substances are added. Then, cells are prepared for lipid peroxidation analysis by using the modified Click-iT^®^ Lipid Peroxidation Detection with the Linoleamide Alkyne (LAA) Kit (Thermo Fisher Scientific, Waltham, MA, USA) protocol. First, the final concentration of 50 μM Click-iT^®^ LAA solution is administered to the cells, and they are incubated for 2 h at room temperature. The cells are washed with 1 × PBS three times to eliminate excess Click-iT^®^ LAA before adding 3.7% formaldehyde in 1 × PBS and incubated at room temperature for 15 min. Afterward, 1 × PBS is used to wash the cells three times. Next, Triton X-100 is diluted to 0.5% in 1 × PBS and added to the cells before incubating for 10 min at room temperature. The cells are then blocked by adding 1% of BSA in 1 × PBS and incubated for half an hour at room temperature. The cells are washed in 1 × PBS twice to remove the BSA entirely. Click-iT^®^ reaction cocktail is prepared for a total volume of 100 μL: 86 μL 1 × Click-iT^®^ reaction buffer, 4 μL CuSO_4_, 0.24 μL Alexa Fluor^®^ 488 azide, and 10 μL 1 × Click-iT^®^ buffer additive. The cells are incubated at room temperature for half an hour in the dark. The cells are washed with 1% BSA in 1 × PBS twice and with 1 × PBS—twice. After that, quantitative and qualitative imaging is performed with a FITC-specific filter set by using a Nikon Eclipse 80i fluorescence microscope, and analysis is conducted using the ImageJ program.

Statistical analysis. Statistical data analysis was performed using the R data analysis program (v4.1.2; R 2021). Position (mean) and dispersion (standard deviation) statistics were used in the work. Each quantitative experiment was performed three times. Data were compared using Student’s *t*-test. Data were considered statistically significant if the determined *p*-value was less than 0.05 (*p* < 0.05). The results of flow cytometry experiments were processed using the FACSDiva (v9.0) and R data analysis programs. The R program packages ggcyto (v1.22.0) and flowCore (v2.6.0) were used for analysis. 

## 3. Results and Discussion

### 3.1. Characterization of Geobacillus *spp.* AgNPs in Sorbitol and YPD Growth Medium

To determine the size and stability of AgNPs obtained using the supernatants of *Geobacillus* spp. 25 and 612 during the experiments, these AgNPs were kept for 12 h in a 1 M sorbitol solution or YPD growth medium. The size distribution results are presented in Table 1.

The obtained results show that the size of most AgNPs in 1 M sorbitol solution is < 100 nm; however, there were reductions (from 99 to 92% for *Geobacillus* sp. 25 AgNPs and from 88 to 87% for *Geobacillus* sp 612 AgNPs) in AgNPs with a diameter of less than 100 nm compared to the results obtained by measuring AgNPs size immediately after the synthesis [10]. A greater reduction in AgNPs less than 100 nm was observed after their incubation in the YPD growth medium (to 52% for *Geobacillus* sp. 25 AgNPs and to 43% for *Geobacillus* sp. 612 AgNPs).

The Zeta potential values of these AgNPs after 12 h of incubation in 1 M sorbitol and YPD growth medium were also measured. The results are presented in Table 2.

Zeta potential value of *Geobacillus* sp. 25 AgNPs shifted to the positive side by 1.25 mV (from −31.28 ± 0.47 mV) in 1 M sorbitol solution; however, the value was the same in the YPD medium compared to the Zeta potential value measured after the synthesis. The Zeta potential value of *Geobacillus* sp. 612 AgNPs shifted to the negative side by 2.70 mV (from −27.43 ± 0.27 mV) in 1 M sorbitol solution and by 0.94 mV in YPD medium compared to Zeta potential values measured after the synthesis [10]. These changes indicate that the components of the solutions are potentially binding to the surface of the AgNPs.

### 3.2. Determination of Silver Nanopaticles MIC and Cell Viability

Determination of the MICs showed that *C. guilliermondii* yeast was more susceptible to the treatment with *Geobacillus* spp. AgNPs compared to *S. cerevisiae.* The MICs for *Geobacillus* sp. 25 AgNPs were 10 μg/mL against *C. guilliermondii* and 50 μg/mL against *S. cerevisiae,* and for *Geobacillus* sp. 612, the MICs were 5 μg/mL and 25 μg/mL, respectively. 

**Figure 1:**
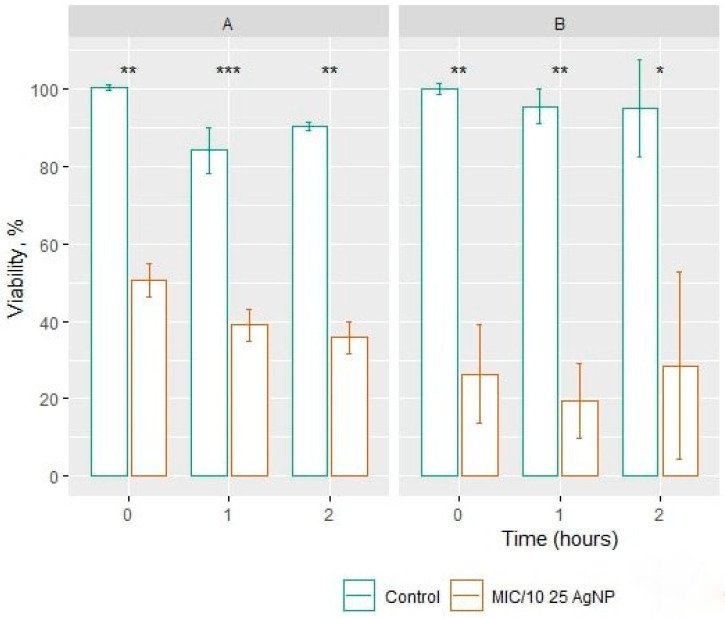
The antifungal effect of *Geobacillus* sp. 25 AgNPs for 1/10 of the MIC against *C. guilliermondii* (**A**) and against *S. cerevisiae* (**B**). *p*-values: <0.05 (*); <0.01 (**); <0.001 (***).

For the time-killing assay, 1/10 of the MIC was chosen. Treatment of *C. guilliermondii* yeast with strain 25 AgNPs (Figure 1A) showed a statistically significant reduction in cell viability already at the start of the incubation (0 h) as compared to the control; cell viability dropped to 49.36 ± 4.36% CFU. After 1 h after incubation, the number of viable cells decreased by 60.97 ± 4.03% and after 2 h—64.26 ± 4.18%. 

Similar results were registered after treatment of *S. cerevisiae* with strain 25 AgNPs (Figure 1B): at the initial time point (0 h), an antifungal effect was also observed, and the number of viable cells decreased by 73.59 ± 12.82%. After 1 h post incubation, the number of viable cells decreased by 80.53 ± 9.61% and after 2 h—71.44 ± 24.14%. 

Cell growth and viability assays indicate whether the stimulus being tested inhibits cell division. The fastest methods include the estimation of the number of CFUs, the droplet test, or estimation of growth in a liquid-nutrient medium. During this work, *Geobacillus* sp. AgNPs’ MICs were determined for the yeasts *S. cerevisiae* and *C. guilliermondii* via strain-induced synthesis of strain 25. The MIC of 25 AgNPs against *S. cerevisiae* was 50 μg/mL and against *C. guilliermondii* was 10 μg/mL. The determined concentrations are similar to those of other researchers (MIC < 70 μg/mL) [22,23]. The results of the yeast cell viability time showed that the antifungal effect of AgNPs occurs already in the first hour of incubation. Likewise, a 10-fold reduction in AgNPs concentration maintained the antifungal effect of AgNPs, and a statistically significant reduction in viability was observed. The results of the clonogenic test show that the viability of yeast cells depends on the AgNP concentration and incubation time. The results of this study allowed the selection of the optimal AgNP concentration and duration of incubation with the microorganism, which can be a critical factor in studying the type of cell death [24]. 

### 3.3. Detection of the Active Caspases

**Figure 2:**
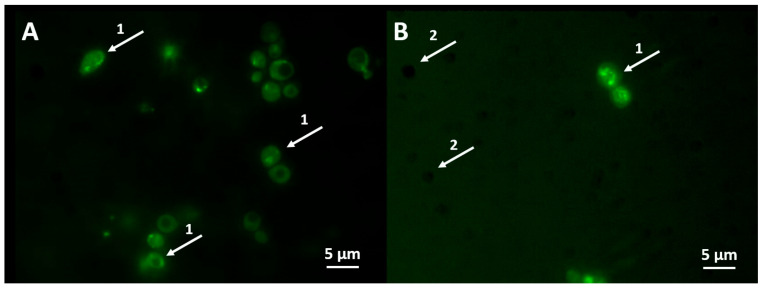
Identification of active caspases in yeast *S. cerevisiae* (**A**) and *C. guilliermondii* (**B**) exposed to MIC concentrations of *Geoabacillus* sp. 25 AgNPs. 1: cells with active caspases are exhibiting green fluorescence and 2: cells without active caspases remaining unstained.

The FITC-VAD-FMK in situ marker was used for the identification of the active caspases. The marker binds to the active caspases and the green fluorescence is detected (Figure 2A,B).

**Figure 3:**
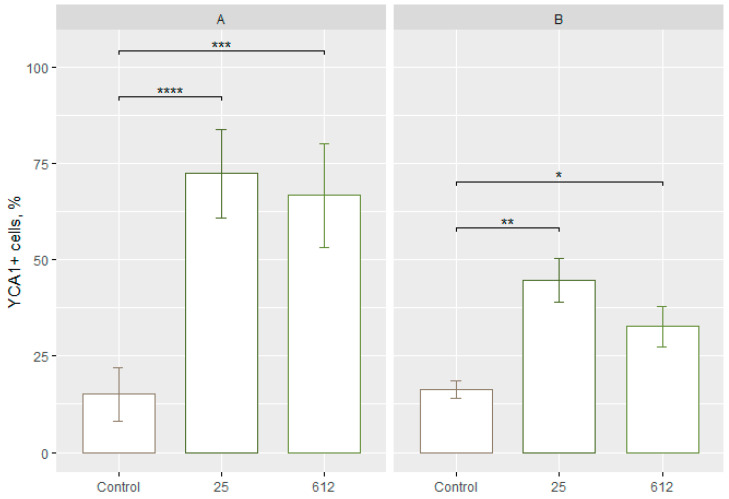
Number (%) of yeast cells with active caspases after 1 h. (**A**) *S. cerevisiae* and (**B**) *C. guilliermondii*. *p*-values: <0.05 (*), <0.01 (**), <0.001 (***), <0.0001 (****). Control—yeast cells unaffected by AgNPs. The numbers at the bottom refer to the *Geobacillus* spp. strain number.

After exposure of yeast *S. cerevisiae* to AgNPs of *Geobacillus* strains 25 and 612 in 1 M sorbitol for 1 h, there were 72.35 ± 8.95% (*p* < 0.0001) and 66.64 ± 10.52% (*p* < 0.001) of yeast cells with active caspases, respectively (Figure 3A). After exposure of yeast *C. guilliermondii* to AgNPs of *Geobacillus* strains 25 and 612 in 1 M sorbitol for 1 h, there were 44.69 ± 5.22% (*p* < 0.01) and 32.71 ± 4.87% (*p* < 0.05) of yeast cells with active caspases, respectively (Figure 3B). The concentrations of AgNPs are 10 μg/mL (*Geobacillus* sp. 25) and 5 μg/mL (*Geobacillus* sp. 612).

The performed study shows that the exposure of yeast cells to AgNPs of strains 25 and 612 in sorbitol for 1 h can activate programmed cell death. The AgNPs of strain 25 induce the activation of active caspases more than the AgNPs of strain 612. Moreover, AgNPs activate caspases to a higher level in *S. cerevisiae* cells compared to *C. guilliermondii*. 

### 3.4. Evaluation of DNA Fragmentation

**Figure 4:**
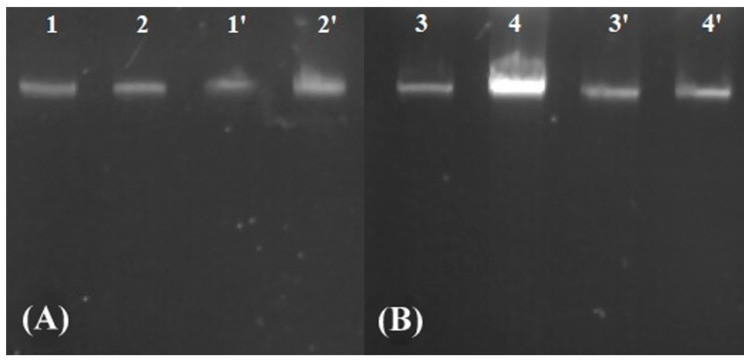
The evaluation of DNA fragmentation in yeasts after exposure to *Geobacillus* sp. 25 AgNPs in 1 M sorbitol. 1, 2: *C. guilliermondii* cells incubated without AgNPs and 1′, 2′: *C. guilliermondii* cells incubated with 1 μg/mL concentration of *Geobacillus* sp. 25 AgNPs (**A**). 3, 4: *S. cerevisiae* cells incubated without AgNPs and 3′, 4′: *S. cerevisiae* cells incubated with 5 μg/mL concentration of *Geobacillus* sp. 25 AgNPs (**B**).

DNA fragmentation was assessed with DNA electrophoresis in agarose gel (Figure 4). The results showed that control and analyzed samples were the same size, the DNA ladders characteristic for apoptosis were not formed, and no DNA fragments of a smaller size were observed. The same results were observed in both conditions: YPD+MIC of AgNPs and sorbitol+1/10 MIC of AgNPs. It is important to mention that apoptosis in yeast can be conducted without DNA fragmentation or single-strand DNA breaks can be observed, which are undetectable with electrophoresis.

### 3.5. Determination of the Cell Membrane Permeability

Increased cell membrane permeability is the marker of late apoptosis or necrosis. The evaluation of membrane permeability was assessed by staining yeast cells with propidium iodide (PI) which, after entering the yeast cell, intercalates with the DNA and exhibits red fluorescence. 

**Figure 5:**
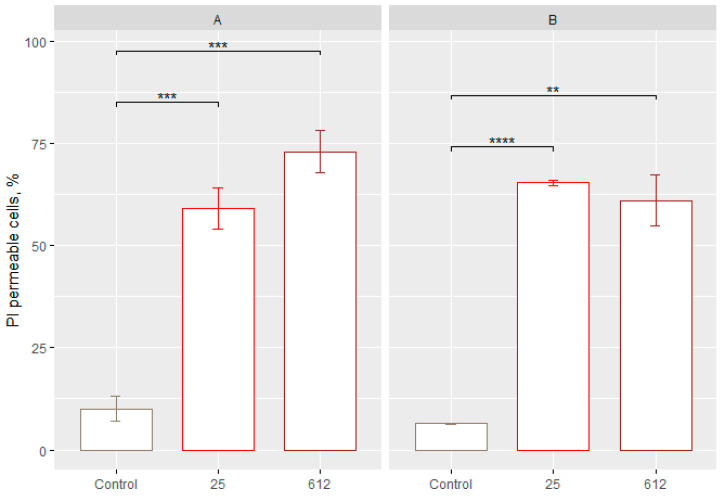
Estimation of the number of cells with permeable membranes in the yeast *S. cerevisiae* after 30 min incubation with 25 and 612 AgNPs (**A**) and *C. guilliermondii* after 30 min incubation with 25 and 612 AgNPs (**B**). *p*-values: <0.01 (**), <0.001 (***), <0.0001 (****). Control—yeast cells unaffected by AgNPs. The numbers at the bottom refer to the *Geobacillus* spp. strain number.

Exposure of yeast *S. cerevisiae* to 25 and 612 AgNPs in sorbitol resulted in a statistically significant increase in the number of cells with permeable membranes (*p* < 0.05), as compared to the control group (Figure 5A). Rates of cells with lost viability were 58.98 ± 4.69% and 72.93 ± 4.80% (Figure 5A). In the case of the yeast *C. guilliermondii*, a statistically reliable change after exposure to 25 AgNPs and 612 AgNPs (Figure 5B) was also detected. After exposure to 25 AgNPs, 65.29 ± 0.51% of cells with permeable membranes were found, and 61.00 ± 5.72% of cells with permeable membranes after exposure to 612 AgNP were found. The concentrations of AgNPs are 10 μg/mL (*Geobacillus* sp. 25) and 5 μg/mL (*Geobacillus* sp. 612). Supplementary Materials Figures S1 and S2 show more detailed flow cytometry results.

The results of this study show that the strongest antifungal effect and the highest number of cells with permeable membranes are evident in the yeast *S. cerevisiae* after 30 min of incubation in sorbitol with 612 AgNPs.

Cell staining with PI is a widely used method to distinguish between live and dead cell populations. PI enters cells with permeable membranes and intercalates with DNA, staining the cells red. This method is often used in conjunction with a clonogenic assay that measures the number of CFUs. In addition, membrane permeability is a marker of late apoptosis (secondary necrosis) or primary necrosis. After staining yeast cells with PI, a statistically reliable number of cells with permeable membranes was found in both yeast cells with all used AgNPs. The PI staining method differentiates cells according to the resulting membrane permeability, which is also associated with loss of viability. In addition, PI staining of cells has the disadvantage that cell membrane integrity can be restored and reversed [25].

### 3.6. Evaluation of ROS Formation

**Figure 6:**
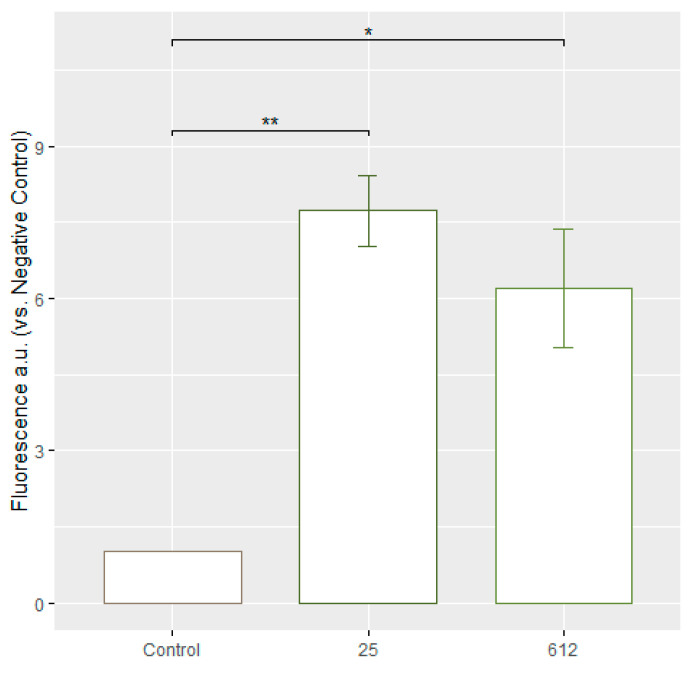
The fold increase in average green fluorescence intensity, relative to the negative control equalized to 1. A fold increase indicates an increase in intracellular ROS levels after treating *C. guilliermondii* yeasts with AgNPs. The numbers beneath each column in the diagram represent the strains of *Geobacillus* bacteria used for synthesizing the applied AgNPs. *p*-values: <0.05 (*); <0.01 (**). Control—yeast cells unaffected by AgNPs. The numbers at the bottom refer to the *Geobacillus* spp. strain number.

To determine the formation of ROS in *C. guilliermondii* after exposure to AgNPs, the DCFDA/H2DCFDA-Cellular ROS Assay Kit (ab113851) was used. DCFDA (2′,7′-dichlorofluorescein diacetate) is a fluorescent dye used to measure the activity of ROS in cells. The dye enters cells and is transformed by cellular esterases into a non-fluorescent compound. When exposed to ROS, this compound is converted into a fluorescent molecule 2′,7′-dichlorofluorescein (DCF) [26]. The ROS formation was evaluated with fluorescent microscopy and flow cytometry. The results were evaluated based on whether the test samples exhibited fluorescence compared to the negative control. No fluorescence was observed in the negative control, while fluorescence was observed and recorded in *C. guilliermondii* cells treated with AgNPs (Figure 6). Obtained results indicate that the AgNPs of both *Geobacillus* spp. strains are capable of inducing increased ROS formation as compared to the control cells. The concentrations of AgNPs are 10 μg/mL (*Geobacillus* sp. 25) and 5 μg/mL (*Geobacillus* sp. 612). Figures S3 and S4 show more detailed flow cytometry results.

In a similar study, *C. albicans* and *S. cerevisiae* cells were treated with AgNPs, and the same ROS-indicating fluoroprobe, DCFDA, was used. The flow cytometry technique was employed to quantify the geometric mean fluorescence intensity (gMFI) in the cellular samples. Significantly higher gMFI, indicating increased levels of ROS, was observed in *C. albicans* cells treated with 2 μg/mL, 20 μg/mL, and 50 μg/mL of AgNPs (5 nm in diameter) compared to the control. Interestingly, no significant increase in gMFI was observed in *S. cerevisiae* [5]. In the article, it is mentioned that a higher concentration of AgNPs leads to greater antifungal activity, as demonstrated with spot assays (comparing 2 µg/mL, 20 µg/mL, and 50 µg/mL). However, when looking at the gMFI values in the diagram, after treating with a concentration of 50 µg/mL of AgNPs, it is evident that it does not result in higher levels of ROS, compared to the concentrations of 2 µg/mL or 20 µg/mL [5]. It was found that the cell death induced in *Candida* with AgNPs may not solely rely on ROS. Other mechanisms such as cell cycle delay, reduction in glucose uptake, and growth suppression were commonly observed in both *C. albicans* and *S. cerevisiae*. However, ROS formation was specifically observed in *C. albicans* cells, indicating potential variations in the antifungal mechanisms of 5 nm AgNPs between these two species [5]. 

### 3.7. Assessment of Lipid Peroxidation

It is well known that ROS formation can be associated with lipid peroxidation [27]. This process was investigated in this study using a Click-iT^®^ Lipid Peroxidation Detection with Linoleamide Alkyne (LAA) Kit. Click-iT^®^ LAA, when added to cells, permeates and is incorporated into their membranes. As the membranes undergo lipid peroxidation, the labeled LAA is oxidized, leading to the production of hydroperoxides. These hydroperoxides then decompose into α, β-unsaturated aldehydes, that can modify proteins in the cells. By using Click-iT^®^ chemistry, these modified proteins, containing the alkyne group, can be detected and analyzed [28].

**Figure 7:**
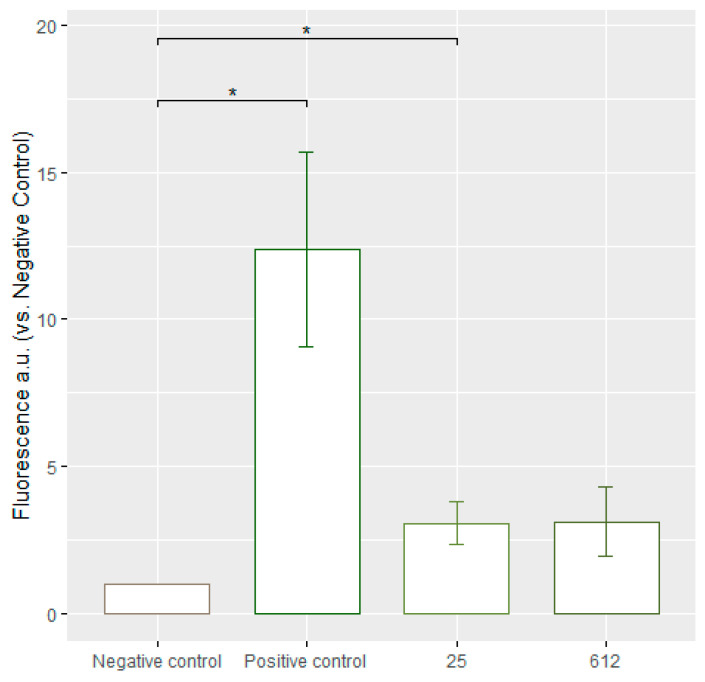
The fold increase in average green fluorescence intensity, relative to the negative control equalized to 1. A fold increase indicates an increase in lipid peroxidation levels after treating *C. guilliermondii* yeasts with AgNPs. The numbers beneath each column in the diagram represent the strains of *Geobacillus* bacteria used for synthesizing the applied AgNPs. *p*-values: * <0.05. Negative control—yeast cells unaffected by AgNPs. The numbers at the bottom refer to the *Geobacillus* spp. strain number; positive control—yeast cells affected with Cumene hydroperoxide.

The 25 and 612 strain AgNPs exhibited similar rates of lipid peroxidation, which was significantly increased as compared to the untreated control (Figure 7). After treatment with AgNPs fluorescence increase was observed up to 3.05 ± 0.72, and 2.49 ± 0.62 in folds, correspondingly. The concentrations of AgNPs are 10 μg/mL (*Geobacillus* sp. 25) and 5 μg/mL (*Geobacillus* sp. 612).

The currently collected data demonstrate a similar trend, which is also observed in other studies with AgNPs obtained by using chemical and physical methods for particle obtainment. *Geobacillus* spp. AgNPs induce statistically significant lipid peroxidation and an increase in lipid peroxidation under the same conditions where ROS production is observed.

Other studies confirm the capability of biosynthesized AgNPs to induce lipid peroxidation. Elbahnasawy and the coauthors showed that *Rothia endophytica* (a strain of bacteria found inside Maize roots) biosynthesized nanoparticles were effective against *C. albicans* and induced lipid peroxidation [29]. The level of MDA, a key marker of lipid peroxidation, was measured to evaluate the extent of lipid degradation. The MDA concentration in the treated cells was noticeably higher, compared to control cells, after a 36 h incubation of *C. albicans* cells. This finding, consistent with the current study, indicates that the AgNPs caused the oxidation of fatty acids in the cell membrane, leading to an increased peroxidation of lipids. The current collected data demonstrate a similar trend, which is also observed in other studies. AgNPs induce statistically significant lipid peroxidation and the increase in lipid peroxidation under the same conditions where ROS production is observed.

## 4. Conclusions

In this research, we showed that *Geobacillus* spp. strains are capable of synthesizing AgNPs, and AgNPs can be successfully used for the elimination of pathogenic yeasts *C. guilliermondii* colonizing human skin, depending on the selected strain. Also, to our knowledge, we have shown for the first time that the apoptotic markers: active caspases and membrane permeabilization, were detected in both yeasts, *S. cerevisiae* and *C. guilliermondii*, after the exposure to *Geobacillus* spp. AgNPs. DNA fragmentation was absent in both yeasts. ROS detection results showed that the highest fold increase in ROS compared to the negative control was observed with the MIC for AgNPs produced by *Geobacillus* sp. 25. Therefore, the lipid peroxidation rate was similar in both cases: after the treatment with *Geobacillus* sp. 25- and *Geobacillus* sp. 612-produced AgNPs. 

## Figures and Tables

**Table 1:** The size distribution (%) of AgNPs incubated for 12 h in 1 M sorbitol solution or YPD medium.

Diameter of AgNPs (nm)	*Geobacillus* sp. 25 AgNPs		*Geobacillus* sp. 612 AgNPs	
	1 M Sorbitol	YPD Medium	1 M Sorbitol	YPD Medium
<10	6	0	3	0
10–20	25	3	18	1
20–30	26	7	20	5
30–40	10	11	9	5
40–50	13	10	11	7
50–60	5	3	8	9
60–70	2	3	5	3
70–80	2	7	3	5
80–90	3	6	4	3
90–100	0	2	6	5
>100	8	48	13	57

**Table 2:** Zeta potential values (mV) of AgNPs incubated for 12 h in 1 M sorbitol solution or YPD medium.

	*Geobacillus* spp. Strain	25	612
Solution			
1 M sorbitol		−30.03 ± 0.05	−30.13 ± 0.28
YPD medium		−30.93 ± 0.21	−28.37 ± 0.14

## Data Availability

The original contributions presented in the study are included in the article and Supplementary Material, further inquiries can be directed to the corresponding author.

## Supplementary Materials

The following supporting information can be downloaded at: https://www.mdpi.com/article/10.3390/jfb15060142/s1, Figure S1: Flow cytometry gating strategy for *C. guilliermondii* stained with propidium iodide (PI); Figure S2: Flow cytometry results of *C. guilliermondii* exposed to *Geobacillus* spp. AgNPs and stained with propidium iodide (PI); Figure S3: Flow cytometry gating strategy for *C. guilliermondii* cells stained with 2′,7′–dichlorofluorescin diacetate (DCFDA); Figure S4: Flow cytometry results of *C. guilliermondii* exposed to *Geobacillus* spp. AgNPs and stained with 2′,7′–dichlorofluorescin diacetate (DCFDA).

## References

[1] Benedetti V.P., Savi D.C., Aluizio R., Adamoski D., Kava-Cordeiro V., Galli-Terasawa L.V., Glienke C. (2016). Analysis of the Genetic Diversity of Candida Isolates Obtained from Diabetic Patients and Kidney Transplant Recipients. Mem. Inst. Oswaldo Cruz.

[2] Lionakis M.S., Drummond R.A., Hohl T.M. (2023). Immune Responses to Human Fungal Pathogens and Therapeutic Prospects. Nat. Rev. Immunol..

[3] Marak M.B., Dhanashree B. (2018). Antifungal Susceptibility and Biofilm Production of *Candida* Spp. Isolated from Clinical Samples. Int. J. Microbiol..

[4] Pfaller M.A., Diekema D.J. (2007). Epidemiology of Invasive Candidiasis: A Persistent Public Health Problem. Clin. Microbiol. Rev..

[5] Lee B., Lee M.J., Yun S.J., Kim K., Choi I.-H., Park S. (2019). Silver Nanoparticles Induce Reactive Oxygen Species-Mediated Cell Cycle Delay and Synergistic Cytotoxicity with 3-Bromopyruvate in Candida Albicans, but Not in Saccharomyces Cerevisiae. Int. J. Nanomed..

[6] Lee N.-Y., Ko W.-C., Hsueh P.-R. (2019). Nanoparticles in the Treatment of Infections Caused by Multidrug-Resistant Organisms. Front. Pharmacol..

[7] Lee Y., Kim J., Oh J., Bae S., Lee S., Hong I.S., Kim S. (2012). Ion-release Kinetics and Ecotoxicity Effects of Silver Nanoparticles. Environ. Toxic. Chem..

[8] Perween N., Khan H.M., Fatima N. (2019). Silver Nanoparticles: An Upcoming Therapeutic Agent for the Resistant Candida Infections. J. Microbiol. Exp..

[9] Alves M.F., Paschoal A.C.C., Klimeck T.D.F., Kuligovski C., Marcon B.H., De Aguiar A.M., Murray P.G. (2022). Biological Synthesis of Low Cytotoxicity Silver Nanoparticles (AgNPs) by the Fungus Chaetomium Thermophilum—Sustainable Nanotechnology. J. Fungi.

[10] Cekuolyte K., Gudiukaite R., Klimkevicius V., Mazrimaite V., Maneikis A., Lastauskiene E. (2023). Biosynthesis of Silver Nanoparticles Produced Using Geobacillus Spp. Bacteria. Nanomaterials.

[11] Giray G., Gonca S., Özdemir S., Isik Z., Yılmaz E., Soylak M., Dizge N. (2023). Novel Extracellular Synthesized Silver Nanoparticles Using Thermophilic *Anoxybacillus Flavithermus* and *Geobacillus Stearothermophilus* and Their Evaluation as Nanodrugs. Prep. Biochem. Biotechnol..

[12] Mohammed Fayaz A., Girilal M., Rahman M., Venkatesan R., Kalaichelvan P.T. (2011). Biosynthesis of Silver and Gold Nanoparticles Using Thermophilic Bacterium Geobacillus Stearothermophilus. Process Biochem..

[13] Grosfeld E.V., Bidiuk V.A., Mitkevich O.V., Ghazy E.S.M.O., Kushnirov V.V., Alexandrov A.I. (2021). A Systematic Survey of Characteristic Features of Yeast Cell Death Triggered by External Factors. J. Fungi.

[14] Arandjelovic S., Ravichandran K.S. (2015). Phagocytosis of Apoptotic Cells in Homeostasis. Nat. Immunol..

[15] Asare N., Instanes C., Sandberg W.J., Refsnes M., Schwarze P., Kruszewski M., Brunborg G. (2012). Cytotoxic and Genotoxic Effects of Silver Nanoparticles in Testicular Cells. Toxicology.

[16] Awashra M., Młynarz P. (2023). The Toxicity of Nanoparticles and Their Interaction with Cells: An in Vitro Metabolomic Perspective. Nanoscale Adv..

[17] Lipke P.N., Ovalle R. (1998). Cell Wall Architecture in Yeast: New Structure and New Challenges. J. Bacteriol..

[18] Navarro-Arias M.J., Hernández-Chávez M.J., Garcia-Carnero L.C., Amezcua-Hernández D.G., Lozoya-Pérez N.E., Estrada-Mata E., Martínez-Duncker I., Franco B., Mora-Montes H.M. (2019). Differential Recognition of Candida Tropicalis, Candida Guilliermondii, Candida Krusei, and Candida Auris by Human Innate Immune Cells. Infect. Drug Resist..

[19] Lastauskienė E., Zinkevičienė A., Girkontaitė I., Kaunietis A., Kvedarienė V. (2014). Formic Acid and Acetic Acid Induce a Programmed Cell Death in Pathogenic Candida Species. Curr. Microbiol..

[20] Suppi S., Kasemets K., Ivask A., Künnis-Beres K., Sihtmäe M., Kurvet I., Aruoja V., Kahru A. (2015). A Novel Method for Comparison of Biocidal Properties of Nanomaterials to Bacteria, Yeasts and Algae. J. Hazard. Mater..

[21] Carmona-Gutierrez D., Bauer M.A., Zimmermann A., Aguilera A., Austriaco N., Ayscough K., Balzan R., Bar-Nun S., Barrientos A., Belenky P. (2018). Guidelines and Recommendations on Yeast Cell Death Nomenclature. Microb. Cell.

[22] Sri Ramkumar S.R., Sivakumar N., Selvakumar G., Selvankumar T., Sudhakar C., Ashokkumar B., Karthi S. (2017). Green Synthesized Silver Nanoparticles from Garcinia Imberti Bourd and Their Impact on Root Canal Pathogens and HepG2 Cell Lines. RSC Adv..

[23] Shanmugaiah V., Harikrishnan H., Al-Harbi N.S., Shine K., Khaled J.M., Balasubramanian N., Kumar R.S. (2015). Facile synthesis of silver nanoparticles using *Streptomyces* sp. VSMGT1014 and their antimicrobial efficiency. Dig. J. Nanomater. Biostruct..

[24] Siddiqi K.S., Husen A., Rao R.A.K. (2018). A Review on Biosynthesis of Silver Nanoparticles and Their Biocidal Properties. J. Nanobiotechnol..

[25] Haase S.B., Reed S.I. (2002). Improved Flow Cytometric Analysis of the Budding Yeast Cell Cycle. Cell Cycle.

[26] LeBel C.P. (1992). Evaluation of the Probe 2’,7’-Dichiorofluorescin as an Indicator of Reactive Oxygen Species Formation and Oxidative Stress. Chem. Res. Toxicol..

[27] Biswas M.d.S., Mano J. (2021). Lipid Peroxide-Derived Reactive Carbonyl Species as Mediators of Oxidative Stress and Signaling. Front. Plant Sci..

[28] Bayır H., Anthonymuthu T.S., Tyurina Y.Y., Patel S.J., Amoscato A.A., Lamade A.M., Yang Q., Vladimirov G.K., Philpott C.C., Kagan V.E. (2020). Achieving Life through Death: Redox Biology of Lipid Peroxidation in Ferroptosis. Cell Chem. Biol..

[29] Elbahnasawy M.A., Shehabeldine A.M., Khattab A.M., Amin B.H., Hashem A.H. (2021). Green Biosynthesis of Silver Nanoparticles Using Novel Endophytic Rothia Endophytica: Characterization and Anticandidal Activity. J. Drug Deliv. Sci. Technol..

