# Supplementary material for: Induction of Apoptosis with Silver Nanoparticles Obtained Using Thermophilic Bacteria
Source: J Funct Biomater. 2024 May 24;15(6):142. doi: 10.3390/jfb15060142 (PMC11205018; doi:10.3390/jfb15060142)
Supplement: Supplementary file 1 [file jfb-15-00142-s001.zip › jfb-3002767-supplementary.pdf]

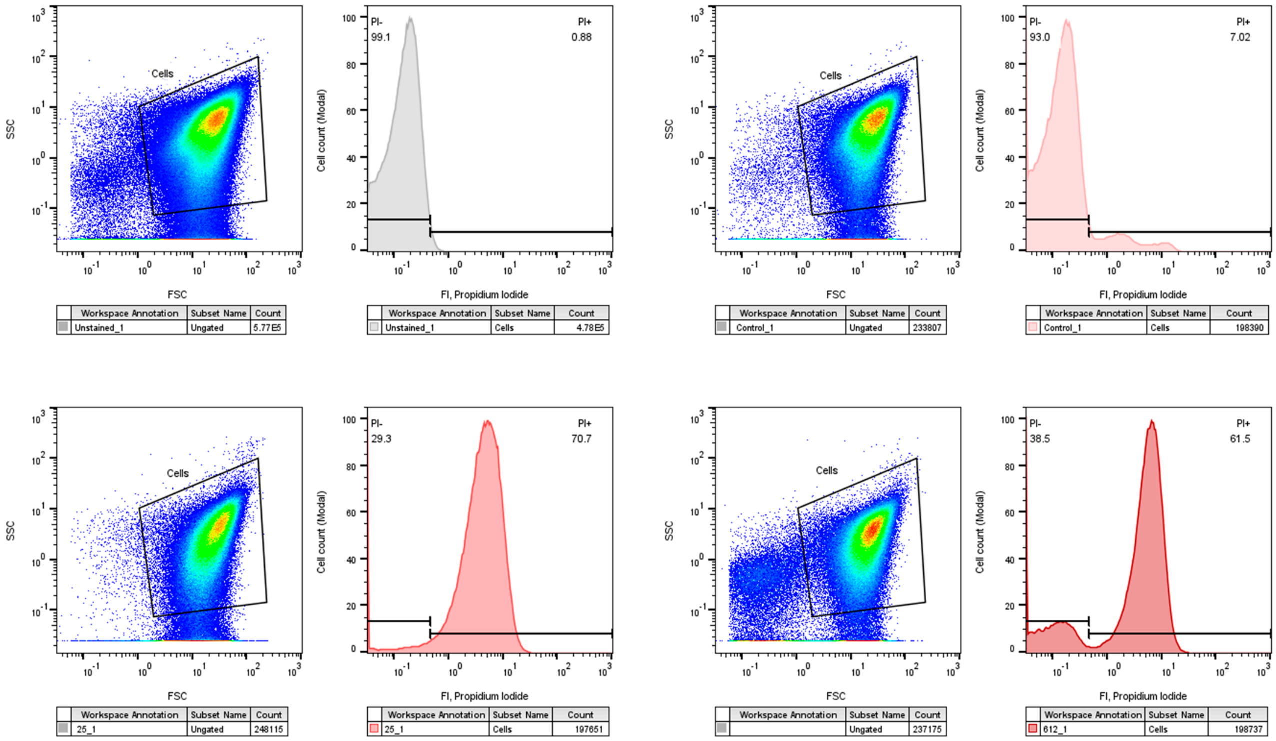

Figure S1. Flow cytometry gating strategy for *C. guilliermondii* stained with propidium iodide (PI). Forward vs side scatter plot showing gate Cells and excluding cell debris and smaller particles. Unstained cells were used as a PI-negative control. In this experiment, at least 100,000 cells were analyzed.

J. Funct. Biomater. **2024**, *15*, 142. https://doi.org/10.3390/jfb15060142 www.mdpi.com/journal/jfb

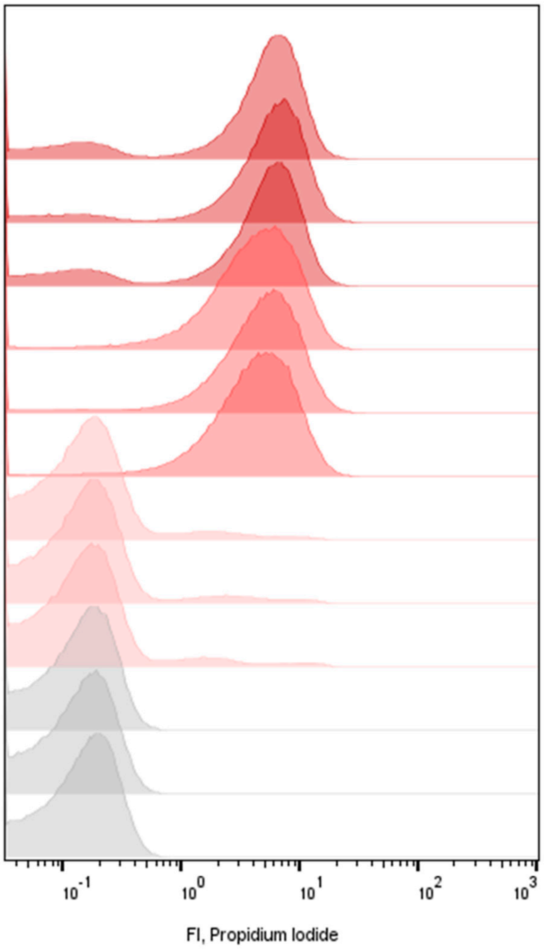

	Workspace Annotation	Subset Name	Count	Freq. of Parent
	612_3	Cells	198885	64.6
	612_2	Cells	198404	64.0
	612_1	Cells	198737	61.5
	25_3	Cells	197476	65.6
	25_2	Cells	197841	65.9
	25_1	Cells	197651	70.7
	Control_3	Cells	198206	6.65
	Control_2	Cells	199021	6.90
	Control_1	Cells	198390	7.02
	Unstained_3	Cells	198519	0.76
	Unstained_2	Cells	198191	0.74
	Unstained_1	Cells	4.78E5	0.88

Figure S2. Flow cytometry results of *C. guilliermondii* exposed to *Geobacillus* spp. AgNPs and stained with propidium iodide (PI). Unstained - *C. guilliermondii* cells used as a PI negative control; Control - *C. guilliermondii* cells unaffected with *Geobacillus* spp. AgNPs and stained with PI; 25 - *C. guilliermondii* cells affected with 10 μg/mL concentration of *Geobacillus* sp. 25 AgNPs, 612 - *C. guilliermondii* cells affected with 5 μg/mL concentration of *Geobacillus* sp. 612 AgNPs. FI - fluorescence intensity.

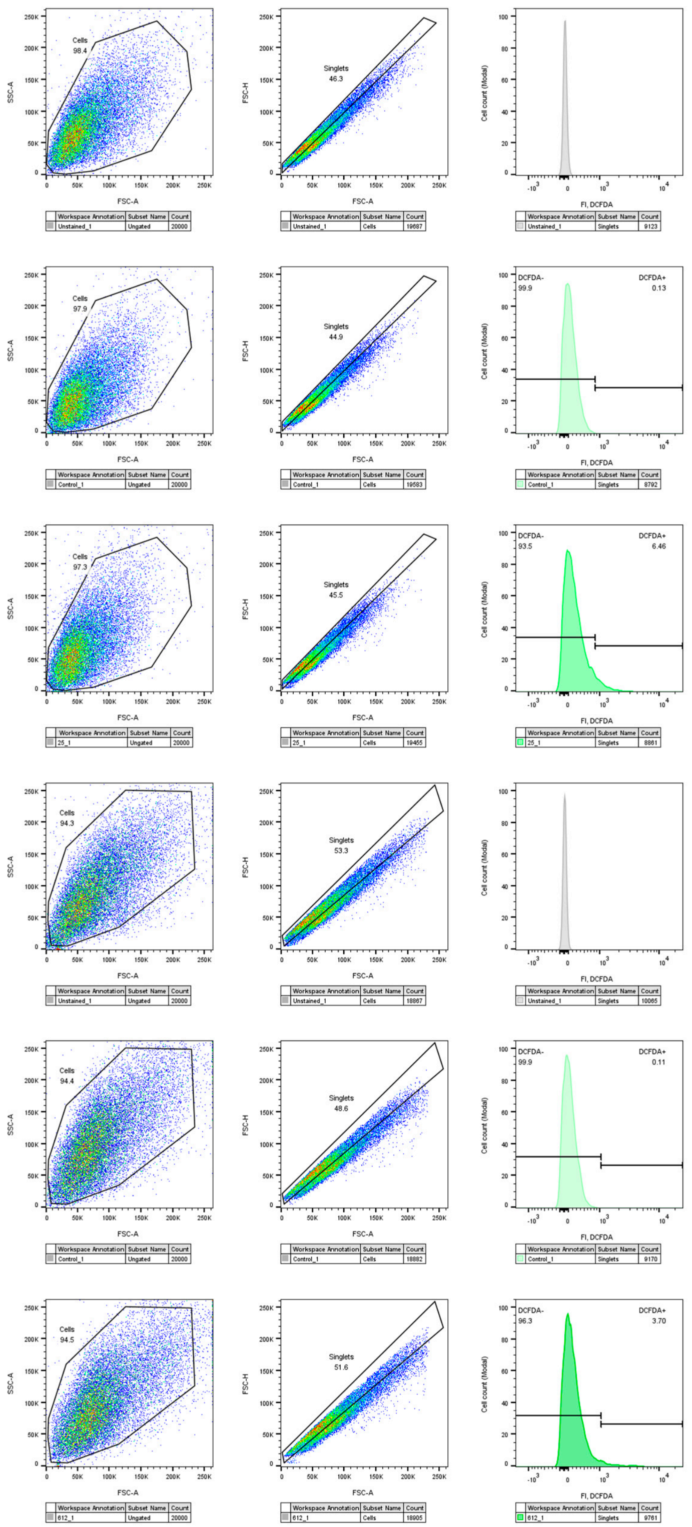

Figure S3. Flow cytometry gating strategy for *C. guilliermondii* cells stained with 2′,7′ –dichlorofluorescin diacetate (DCFDA). Side vs forward scatter plot showing gate Cells and excluding cell debris and small particles. FSC-A vs FSC-H scatter plot showing Singlets. Unstained cells were used as a DCFDA negative control. Control - *C. guilliermondii* cells unaffected with *Geobacillus* spp. AgNPs and stained with DCFDA were used as a negative control. Everything above this level of fluorescence intensity was considered positive in samples affected with *Geobacillus* spp. AgNPs.

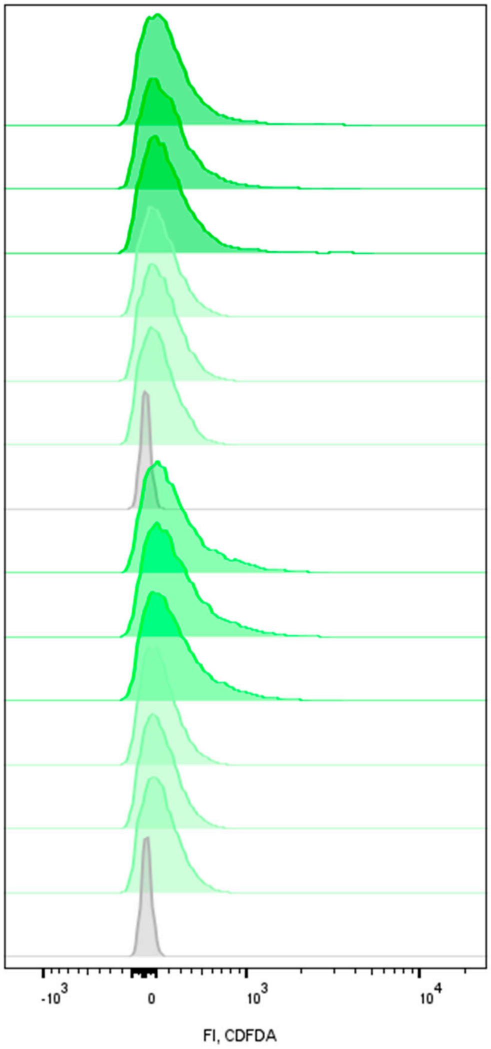

	Workspace Annotation	Subset Name	Count	Freq. of Parent
	612_3	Singlets	9820	3.64
	612_2	Singlets	9813	3.17
	612_1	Singlets	9761	3.70
	Control_3	Singlets	9687	0.13
	Control_2	Singlets	9411	0.15
	Control_1	Singlets	9170	0.11
	Unstained_1	Singlets	10065	n/a
	25_3	Singlets	9007	7.33
	25_2	Singlets	9036	7.19
	25_1	Singlets	8861	6.46
	Control_3	Singlets	9053	0.18
	Control_2	Singlets	8921	0.16
	Control_1	Singlets	8792	0.13
	Unstained_1	Singlets	9123	n/a

Figure S4. Flow cytometry results of *C. guilliermondii* exposed to *Geobacillus* spp. AgNPs and stained with 2′,7′ –dichlorofluorescin diacetate (DCFDA). Unstained - *C. guilliermondii* cells used as a DCFDA negative control; Control - *C. guilliermondii* cells unaffected with *Geobacillus* spp. AgNPs and stained with DCFDA were used as negative control; 25 - *C. guilliermondii* cells affected with 10 μg/mL concentration of *Geobacillus* sp. 25 AgNPs, 612 - *C. guilliermondii* cells affected with 5 μg/mL concentration of *Geobacillus* sp. 612 AgNPs. FI – fluorescence intensity.
